# Correlates of insulin clearance in apparently healthy non-obese Japanese men

**DOI:** 10.1038/s41598-017-01469-x

**Published:** 2017-05-03

**Authors:** Hideyoshi Kaga, Yoshifumi Tamura, Kageumi Takeno, Saori Kakehi, Takashi Funayama, Yasuhiko Furukawa, Miho Nishitani-Yokoyama, Kazunori Shimada, Hiroyuki Daida, Shigeki Aoki, Adria Giacca, Akio Kanazawa, Ryuzo Kawamori, Hirotaka Watada

**Affiliations:** 1 Department of Metabolism & Endocrinology, Juntendo University Graduate School of Medicine, Tokyo, Japan; 2 Sportology Center, Juntendo University Graduate School of Medicine, Tokyo, Japan; 3 Department of Cardiology, Juntendo University Graduate School of Medicine, Tokyo, Japan; 4 Department of Radiology, Juntendo University Graduate School of Medicine, Tokyo, Japan; 5 Center for Therapeutic Innovations in Diabetes, Juntendo University Graduate School of Medicine, Tokyo, Japan; 6 Center for Identification of Diabetic Therapeutic Targets, Juntendo University Graduate School of Medicine, Tokyo, Japan; 7 Departments of Physiology and Medicine, Institute of Medical Science and Banting and Best Diabetes Centre, University of Toronto, Toronto, Canada

## Abstract

Hyperinsulinemia observed in obese subject is caused at least in part by low metabolic clearance rate of insulin (MCRI). However, the determinants of MCRI in non-obese subjects are not fully understood. To investigate the correlates of MCRI in healthy non-obese men (BMI <25 kg/m^2^), we studied 49 non-obese Japanese men free of cardiometabolic risk factors. Using a 2-step hyperinsulinemic euglycemic clamp, we evaluated MCRI and insulin sensitivity. We also calculated the rate of glucose disappearance (Rd) during the clamp and muscle insulin sensitivity was defined as Rd/steady state serum insulin (SS_SI_) at the second step. Based on the median value of MCRI, the subjects were divided into the low- and high-MCRI groups. Subjects of the low-MCRI group had significant impairment of muscle insulin sensitivity, although Rd levels were comparable between the two groups, probably due to elevated SS_SI_ in the low-MCRI group. Subjects of the low-MCRI group had higher total body fat content and lower VO_2peak_ and showed no deterioration of cardiometabolic risk factors. Our results suggest that low MCRI may be early change to maintain glucose uptake and metabolic status in the face of slight impairment of muscle insulin sensitivity caused by increased adiposity and lower fitness level.

## Introduction

Obesity and the associated metabolic abnormalities are worldwide health problems. Insulin resistance and coexistent hyperinsulinemia are important in the pathogenesis of type 2 diabetes mellitus (T2DM) and metabolic abnormalities in obese subjects^1^. Insulin resistance can be compensated by hyperinsulinemia; the latter is not only due to enhanced insulin secretion, but low insulin clearance. In fact, the latter was found to be associated with increased serum insulin level in overweight and obese subjects^2, 3^. In addition, low insulin clearance correlates with metabolic abnormalities^4^ and insulin resistance^5, 6^ in nondiabetic overweight and obese subjects. Thus, low insulin clearance seems to be a feature of obese subjects with insulin resistance and metabolic syndrome^4^.

Several studies demonstrated changes in insulin clearance even in non-obese subjects and that such change could be the initial event predating insulin resistance and obesity^7, 8^. In animal studies, healthy dogs fed high-fat diet for 12 weeks showed moderate accumulation of visceral fat without changes in body weight and mild insulin resistance accompanied by hyperinsulinemia^7^. In this setting, decreased hepatic insulin clearance rather than increased insulin secretion was the major determinant of the hyperinsulinemia^7, 9^. Similarly, a human study also demonstrated that intentional weight gain within the normal range (from BMI of 21.8 to 23.8 kg/m^2^) resulted in impaired insulin sensitivity and hyperinsulinemia and that the hyperinsulinemia was mainly due to reduced insulin clearance rather than enhanced insulin secretion^8^. Thus, insulin clearance can decrease during modest weight gain even when it is within the normal range and may be secondary to initial insulin resistance or can be the initial factor causing impaired insulin sensitivity and adiposity. However, impaired insulin clearance observed in the above human study in non-obese individuals^8^ occurred when body weight was acutely increased by overfeeding (6.2 kg over a 10-week period) and glucose disposal decreased. To our knowledge, there is little or no information on the factors that determine insulin clearance in apparently healthy non-obese subjects who do not undergo dietary intervention. In addition, insulin sensitivity could be impaired in muscle and liver in non-obese subjects, independently^10^; however, it has also not been determined which site of insulin resistance is more associated with changes in insulin clearance.

Based on the above background, the present study was designed to identify the clinical correlates of insulin clearance in apparently healthy non-obese subjects. For this purpose, we recruited healthy non-obese (<25 kg/m^2^) Japanese men without any cardiometabolic risk factors to measure insulin clearance and insulin resistance in muscle and liver by a 2-step euglycemic hyperinsulinemic clamp. The results were analyzed for the association of various metabolic parameters with insulin clearance.

## Results

### Anthropometric characteristics of low- and high- metabolic clearance rate of insulin (MCRI) groups

Table 1 summarizes the clinical characteristics of the study subjects. The mean values of the entire group for cardiometabolic risk factors, fasting serum insulin (FSI), homeostasis model assessment of insulin resistance (HOMA-IR) and other parameters were within the normal ranges. For further analysis, we divided the subjects into low-MCRI group (n = 25) and high-MCRI group (n = 24), based on the median value of MCRI [594.1 (561.3–663.2) ml/min/m^2^]. As shown in Table 1, subcutaneous fat area and total fat content were significantly higher, while visceral fat area tended to be higher, in the low-MCRI group than the high-MCRI group. In addition, VO_2peak_ was significantly lower in the low-MCRI group than the high-MCRI group. Although fasting C-peptide was comparable between the two groups, the FSI was significantly higher in the low-MCRI group than the high-MCRI group probably due to the difference in insulin clearance between the two groups. While FPG levels were comparable between the two groups, HOMA-IR was higher in the low-MCRI group than the high-MCRI group. Similarly, during oral glucose tolerance test (OGTT), the insulin level was higher and MATSUDA index was lower in the low-MCRI group compared with the high-MCRI group. The above data suggest that subjects of the low-MCRI group were characterized by moderate fat accumulation, low fitness level, higher insulin level, and impaired insulin sensitivity, compared to those of the high-MCRI group. Although these features seem to be associated with deterioration in cardiometabolic risk factors, such as hypertension, dyslipidemia and hyperglycemia, even in non-obese subjects with BMI of 23–25 kg/m^2^ ^10^, the cardiometabolic risk factors were comparable between the two groups (Table 1).

**Table 1:** Clinical characteristics of the Low MCRI group and the High MCRI group.

	Total	Low-MCRI	High-MCRI	p value
n	49	25	24	
MCRI (ml/min per m^2^)	594.1 (561–663)	561.3 (534–576)	668.8 (616–715)	**<0.001**
Age (years)	40 (36–45)	40 (35–46)	41 (37–44)	0.682
BMI (kg/m^2^)	23.1 ± 1.0	23.4 ± 0.9	22.9 ± 1.1	0.085
Systolic blood pressure (mmHg)	118.6 ± 7.0	117.8 ± 7.0	119.5 ± 7.0	0.403
Diastolic blood pressure (mmHg)	75.4 ± 5.7	74.3 ± 5.7	76.5 ± 5.7	0.182
Fasting plasma glucose (mg/dl)	93.2 ± 6.8	93.6 ± 7.6	92.9 ± 6.0	0.714
Fasting serum insulin (μU/mL)	4.9 ± 2.1	5.70 ± 1.76	4.05 ± 2.02	**0.004**
Fasting serum C-peptide (ng/ml)	1.23 ± 0.38	1.33 ± 0.33	1.14 ± 0.40	0.077
AUC-glucose during OGTT (mg·min/dl·10^3^)	21.3 ± 2.9	21.7 ± 2.9	20.9 ± 2.8	0.294
AUC-insulin during OGTT (μU·min/ml·10^3^)	5.2 ± 2.8	6.1 ± 2.8	4.3 ± 2.6	**0.020**
HOMA-IR	1.13 ± 0.49	1.32 ± 0.45	0.93 ± 0.46	**0.004**
MATSUDA index	6.7 (4.2–10.9)	5.5 (3.7–7.7)	8.7 (6.1–12.1)	**0.008**
Free fatty acid (μEq/L)	335 ± 105	352 ± 97	318 ± 112	0.261
Triglyceride (mg/dl)	108 ± 46	109 ± 34	107 ± 57	0.899
High density lipoprotein cholesterol (mg/dl)	59.0 ± 13.8	58.9 ± 13.8	59.1 ± 13.8	0.951
HbA1c (%)	4.9 ± 0.2	4.8 ± 0.2	4.9 ± 0.2	0.566
High molecular weight-adiponectin (ng/ml)	1.82 ± 1.21	1.68 ± 1.27	1.97 ± 1.15	0.416
C reactive protein (ng/ml)	177 (125–490)	259 (156–502)	148 (89–379)	0.348
Intramyocellular lipid in TA (S-fat/Cre)	3.2 ± 1.9	3.4 ± 1.7	3.0 ± 2.1	0.525
Intramyocellular lipid in SOL (S-fat/Cre)	12.8 ± 6.8	14.2 ± 7.0	11.3 ± 6.4	0.150
Intra hepatic lipid (%)	1.0 (0.1–2.0)	1.0 (0.1–2.7)	0.7 (0.2–1.6)	0.085
Total body fat content (%)	20.1 ± 5.0	22.2 ± 4.5	18.0 ± 4.6	**0.002**
Abdominal visceral fat area (cm^2^)	75.3 ± 28.0	82.1 ± 28.6	68.3 ± 26.0	0.085
Abdominal subcutaneous fat area (cm^2^)	106 ± 40	125 ± 33	87.6 ± 37.9	**0.001**
VO_2peak_ (ml/kg per min)	36.0 ± 7.0	33.7 ± 5.8	38.5 ± 7.5	**0.015**
Daily Physical Activity (METs · h)	4.98 ± 2.24	4.69 ± 1.35	5.28 ± 2.89	0.365
Daily alcohol intake (g/day)	20.0 ± 24.8	19.6 ± 19.7	20.4 ± 29.7	0.919

Data are mean ± SD or median (interquartile range).

MCRI; metabolic clearance rate for serum insulin, HOMA-IR; homeostasis model assessment of insulin resistance, TA; tibialis anterior muscle, S-fat; methylene signal intensity, VO_2peak_; peak oxygen consumption, AUC; area under the curve, OGTT; oral glucose tolerance test.

### Insulin sensitivity in muscle and liver evaluated by glucose clamp

We evaluated insulin sensitivity in muscle and liver by the gold standard method; the 2-step hyperinsulinemic euglycemic clamp (Table 2). The steady-state serum insulin (SS_SI)_ level during glucose clamp was higher in the low-MCRI group at the first and second steps compared with the high-MCRI group. Nevertheless, the rate of disappearance of glucose (Rd) levels at both the first and second steps were comparable between the two groups, suggesting impaired insulin sensitivity in peripheral tissue (mainly muscle) in subjects of the low-MCRI group. Since Rd is known to be enhanced in parallel with serum insulin concentration^11^, we divided Rd by SS_SI_ at the second step and used the product as an index of muscle insulin sensitivity^12^. As shown in Table 2, muscle insulin sensitivity (Rd/SS_SI_ at the second step) was significantly lower in the low-MCRI group than in the high-MCRI group. These data suggest impaired muscle insulin sensitivity in the low-MCRI group; although Rd during glucose clamp was maintained by increased SS_SI_ due to decreased MCRI.

**Table 2:** Euglycemic hyper-insulinemic clamp data in the Low-MCRI and High-MCRI groups.

	Total	Low-MCRI	High-MCRI	p value
n	49	25	24	
SS_SI_ at first step (μU/mL)	19.2 ± 3.5	20.6 ± 2.7	17.8 ± 3.8	**0.005**
SS_SI_ at second step (μU/mL)	35.1 ± 7.8	39.8 ± 3.4	32.9 ± 4.3	**<0.001**
Basal EGP (mg/m^2^·min^−1^)	80.6 ± 6.4	78.6 ± 5.1	82.5 ± 7.1	**0.032**
%reduction of EGP at first step (%)	71.7 ± 15.8	74.9 ± 15.4	68.3 ± 15.8	0.144
%reduction of EGP at second step (%)	89.5 (82.2–93.9)	87.1 (84.1–93.0)	89.7 (78.2–96.4)	0.274
%reduction of EGP/SS_SI_ at first step (%/μU·ml^−1^)	3.7 ± 1.0	3.7 ± 0.9	3.7 ± 1.2	0.967
Rd at first step (mg/kg FFM·min^−1^)	4.4 ± 1.2	4.2 ± 1.1	4.6 ± 1.3	0.262
Rd at second step (mg/kg FFM·min^−1^)	8.6 ± 2.0	8.1 ± 1.7	9.0 ± 2.2	0.098
Rd /SS_SI_ at second step (mg/kg FFM·min^−1^ μU^−1^·ml)	0.22 (0.19–0.29)	0.20 (0.18–0.23)	0.29 (0.22–0.33)	**<0.001**

Data are mean ± SD or median (interquartile range).

SS_SI_; steady state serum insulin, EGP; endogenous glucose production, Rd; rate of disappearance.

Similar to Rd, endogenous glucose production (EGP) is also known to be suppressed in accordance with insulin concentration at low insulin levels (~20 μU/ml)^11^. Accordingly, we divided the %reduction of EGP by SS_SI_ at the first step and used it as an index of hepatic insulin sensitivity^13^. As shown in Table 2, hepatic insulin sensitivity was comparable between the two groups. In addition, the basal EGP level was significantly lower in the low-MCRI group than in the high-MCRI group, probably due to higher insulin level in the low-MCRI group.

### Correlations between MCRI and other parameters

To further investigate the association of MCRI with various metabolic parameters, correlation analysis was performed between MCRI and several metabolic parameters (Table 3). In this analysis, parameters with *p* values less than 0.1 shown in Table 1 and glucose clamp data shown in Table 2 were selected. MCRI correlated significantly with body fat content and subcutaneous fat area (SFA), but not with the visceral fat area (VFA). In addition, MCRI correlated significantly with both FSI and AUC-insulin during OGTT, again suggesting the contribution of MCRI to hyperinsulinemia at fasting and during OGTT. The results also showed a significant correlation between MCRI and VO_2peak_.

**Table 3:** Results of univariate regression analysis of MCRI in apparently healthy subjects.

	MCRI	
	*r*	*p* value
BMI	−0.22	0.130
Fasting serum insulin	−0.46	**0.001**
Fasting serum C-peptide	−0.20	0.164
AUC-insulin during OGTT	−0.36	**0.010**
HOMA-IR	−0.43	**0.002**
Matsuda Index	0.40	**0.005**
Total body fat content	−0.36	**0.010**
Visceral fat area	−0.25	0.079
Subcutaneous fat area	−0.40	**0.004**
Intrahepatic lipid	−0.16	0.304
VO_2peak_	0.30	**0.039**
SS_SI_ at 1^st^ step	−0.49	**<0.001**
SS_SI_ at 2^nd^ step	−0.84	**<0.001**
Basal EGP	0.32	**0.024**
%reduction of EGP at first step	−0.39	**0.007**
%reduction EGP at second step	−0.09	0.522
%reduction of EGP/SS_SI_ at first step	−0.12	0.430
Rd at first step	0.14	0.354
Rd at second step	0.17	0.231
Rd /SS_SI_ at second step	0.54	**<0.001**

HOMA-IR; homeostasis model assessment of insulin resistance, AUC; area under the curve, OGTT; oral glucose tolerance test, VO_2peak_; peak oxygen consumption, EGP; endogenous glucose production, Rd; rate of disappearance, SS_SI_; Steady state serum insulin.

MCRI correlated negatively with basal EGP and %reduction of EGP at the first step, but not with hepatic insulin sensitivity (%reduction of EGP/SS_SI_ at the first step). In contrast, MCRI did not correlate with Rd at the first and second steps, but correlated significantly with muscle insulin sensitivity (Rd/SS_SI_ at the second step).

## Discussion

We investigated the clinical correlates of insulin clearance in non-obese Japanese men free of cardiometabolic risk factors. The results showed that subjects of the low-MCRI group exhibited moderate fat accumulation, low fitness level, high insulin level and impaired insulin sensitivity in muscle, but not in liver, compared with the high-MCRI group. In subjects of the low-MCRI group, the Rd level during the glucose clamp was maintained by elevated SS_SI_ level and no deterioration of cardiometabolic risk factors was observed.

Previous studies suggested that low MCRI values were associated with obesity^14, 15^, insulin resistance^5, 6^ and cardiometabolic risk factors^4, 16, 17^. However, there is little or no information on the factors that determine insulin clearance in apparently healthy non-obese subjects. This is in part because sequential 2-step hyperinsulinemic euglycemic clamp study, the gold standard method to precisely evaluate hepatic and muscle insulin sensitivity, has not been applied previously for a large number of apparently healthy non-obese subjects. The present study demonstrated impaired muscle insulin sensitivity (Rd/SS_SI_ at the second step) in the low-MCRI group compared with the high-MCRI group; however, hepatic insulin sensitivity and cardiometabolic risk factors were comparable between the two groups. In addition, previous studies demonstrated that decreased MCRI is observed in obese subjects with decreased glucose utilization during glucose clamp^14, 15^; however in the present study, despite impaired muscle insulin sensitivity in subjects of the low-MCRI group, the Rd at the second step during the glucose clamp test did not decrease probably due to elevated serum insulin concentration induced by low MCRI (Table 2). These data suggest that MCRI is well correlated to insulin resistance in muscle, but not in liver. In addition, the data also suggest that the insulin resistance in muscle seen in the low-MCRI group was mild, thus increased serum insulin concentrations induced by lower MCRI was sufficient to maintain the Rd level. Thus, one can hypothesize that in non-obese apparently healthy subjects, low MCRI is a compensatory mechanism to maintain muscle glucose uptake and metabolic status in the face of minuscule impairment of insulin sensitivity in muscle.

On the other hand, it has been shown that low MCRI could be a primary change for insulin resistance. Indeed, in several animal models impaired insulin clearance induced an insulin resistant state^18, 19^. For example, carcinoembryonic antigen-related cell adhesion molecule 1 (CEACAM1) promotes receptor-mediated insulin uptake and degradation in hepatocytes^18, 19^. Liver-specific inactivation or global null-mutation of CEACAM1 impaired hepatic insulin clearance and induced chronic hyperinsulinemia, resulting in insulin resistance and adiposity^18, 19^. In addition, high-fat diet diminished CEACAM1 expression and induced impaired insulin clearance, hyperinsulinemia and insulin resistance in C57/BL6J mice, and these effects were counteracted by liver-specific inducible CEACAM1 expression^19^. These data suggest that impaired insulin clearance could be the primary culprit in insulin resistant states. Furthermore, low MCRI can also induce insulin resistance via hyperinsulinemia, such as that seen after insulin-stimulated fat accumulation, downregulation of insulin receptor^20^ and by post receptor mechanisms such as serine-threonine phosphorylation by insulin-stimulated Erk or S6K^21^. Thus, it is still unclear whether low MCRI is a consequence of slightly impaired insulin sensitivity or a cause of insulin resistance and adiposity. The existence of a vicious cycle between insulin resistance and low insulin clearance renders the search for the primary defect difficult.

Although the mechanisms of how low MCRI can cause insulin resistance have been reported as mentioned above, the mechanisms of how insulin resistance can cause a decrease in MCRI are less known. One possibility is that certain factors that influence both MCRI and muscle insulin sensitivity may mediate a link between MCRI and insulin sensitivity in muscle. For example, previous studies showed that free fatty acids (FFA) do not only induce muscle insulin resistance^22^, but also decrease MCRI^23, 24^. It has been also suggested that FFA is a major source of intrahepatic lipid (IHL) accumulation^25^ and fatty liver is associated with decreased MCRI^26^. Thus, FFA and IHL accumulation can be key factors linking MCRI to insulin resistance; however, both FFA and IHL accumulation were not associated with MCRI in the present study. Another possibility is that muscle activity by aerobic exercise may mediate the association between muscle insulin sensitivity and MCRI. For example, one-year aerobic exercise (jogging) enhanced muscle insulin sensitivity in healthy young men^27^; however, glucose infusion rate during glucose clamp was not increased as anticipated, because aerobic exercise greatly increased MCRI by 87% and reduced SS_SI_ during glucose clamp. A recent animal study indicated that one bout of exercise increased insulin clearance in parallel with increased expression of insulin degrading enzyme in the liver and skeletal muscle^28^. Taken together, we hypothesize that aerobic exercise enhances both muscle insulin sensitivity and MCRI and mediates the link between these factors. Given that aerobic exercise enhances fitness level (VO_2peak_)^29^, our observation that MCRI correlated significantly with VO_2peak_ is in agreement with this hypothesis.

The present study has several limitations. First, we did not perform a pancreatic clamp to completely suppress endogenous insulin secretion throughout the experiment (basal period and clamp). To take this fact into consideration, we adjusted the calculation of MCRI according to the suppression of C-peptide level using a previously published method^30, 31^. Second, we recruited only Japanese in this study, thus our results may not be applicable to other ethnic groups. In fact, previous reports suggested that insulin clearance is lower in South Asians than East Asians and Caucasians^32^. Third, we included men only in this study though metabolism and fat distribution are known to be different between men and women. In addition, we only included middle aged men (30–50 y.o.). Previous study reported decreased MCRI in elderly compared with young^33^, while age was not associated with MCRI in the present study. Finally and most importantly, the present study is cross-sectional and thus cannot address causality.

In conclusion, in non-obese apparently healthy Japanese men, low MCRI was associated with impaired muscle insulin sensitivity, lower fitness level and increased adiposity. However, the Rd level was maintained during glucose clamp test through elevated steady state serum insulin, and there was no increase in cardiometabolic risk factors. These data suggest that in non-obese apparently healthy men, low MCRI could be viewed as an early change observed with modest insulin resistance and could be a compensatory phenomenon to maintain glucose uptake and metabolic status in the face of slightly impaired insulin sensitivity in muscle. However, which of the two factors occurs first, insulin resistance or decreased insulin clearance, is a question that requires further longitudinal studies.

## Research Design and Methods

### Study subjects

The MCRI was assessed in participants of the Sportology Center Core Study, a prospective observational study to support hypothesis-driven, hypothesis-generating research on the underlying mechanisms of metabolic abnormalities in non-obese subjects^10^. In the study, we recruited non-diabetic Japanese men with BMI of 21 to 27.5 kg/m^2^ (≥21.0 to <27.5 kg/m^2^), aged between 30 and 50 years. The study participants were described in detail previously^10^. To assess the role of MCRI in apparently healthy non-obese men, we selected from the cohort those with BMI of 21 to 25 kg/m^2^ (≥21.0 to <25.0 kg/m^2^) who were free of cardiometabolic risk factors [based on the definition of metabolic syndrome in Japan^34^, we used three cardiometabolic risk factors, including hyperglycemia (fasting plasma glucose (FPG) ≥110 mg/dl), dyslipidemia (triglycerides (TG) ≥150 mg/dl and/or high-density lipoprotein-C (HDL-C) <40 mg/dl) and hypertension (systolic blood pressure (SBP) ≥130 mmHg and/or diastolic BP (DBP) ≥85 mmHg)] (n = 52). Among the selected group, the values of C-peptide were not available in 3 participants, who were, accordingly, excluded from the group. Thus, in the present study, we analyzed the baseline data of 49 men (Table 1). All participants gave written informed consent to the study, which was approved by the ethics committee of Juntendo University. This study was carried out in accordance with the principles outlined in the Declaration of Helsinki.

### Study design

The design of the Sportology Center Core Study was described previously in detail^10^. Briefly, after the screening session, all participants visited our institute three times for baseline evaluation. At the first or second visit, each participant underwent OGTT or peak oxygen uptake test as described previously^10, 35^. The participants were instructed to quit regular exercise for 10 days before the third visit and the mean daily physical activity level was evaluated over 7 days with an accelerometer (Lifecorder; Suzuken, Nagoya, Japan). Then, each participant was asked to maintain daily physical activity at mean daily physical activity level ±10% during the last 3 days, which was monitored by an accelerometer.

On the day of the experiment, we measured intramyocellular lipid (IMCL) and IHL by ^1^H-magnetic resonance spectroscopy (MRS) after an overnight fast. Total body fat content and fat-free mass (FFM) were measured by the bioimpedance method (InBody; Biospace, Tokyo)^36^. Furthermore, VFA and SFA were also estimated by magnetic resonance imaging (MRI). Then, euglycemic hyperinsulinemic clamp was performed to measure insulin sensitivity in muscle and liver (see below). Surrogate markers of insulin resistance [i.e., HOMA-IR and Matsuda index] were calculated as described previously^10, 37^.

### Euglycemic hyperinsulinemic glucose clamp

The participants were instructed to consume a weight-maintaining standard diet on the 3 days immediately preceding the clamp study. In addition, they were asked to refrain from alcohol the day before the clamp study. After an overnight fast, a two-step euglycemic hyperinsulinemic glucose clamp study was performed with an artificial endocrine pancreas (STG 22; Nikkiso, Shizuoka, Japan)^10^. Briefly, after securing an intravenous cannula in the forearm, a bolus dose [200 mg/m^2^ body surface area (BSA)] of [6,6-^2^H_2_]glucose (Cambridge Isotope Laboratories, Tewksbury, MA) was injected intravenously, followed by constant infusion of 2 mg/m^2^ BSA per min for 3-h (−180 to 0 min) to measure fasting EGP^38^. This was followed by primed insulin infusion (40 mU/m^2^ per min followed by 20 mU/m^2^ per min; each lasting 5 min) and continuous insulin infusion at 10 mU/m^2^ per min for 3 hours (first step) (0 to 180 min). In the second step of the clamp, after a priming insulin infusion (80 mU/m^2^ per min followed by 40 mU/m^2^ per min; each lasted 5 min), insulin was infused continuously at 20 mU/m^2^ per min for 3 hours (180 to 360 min). The infusion of [6,6-^2^H_2_]glucose was decreased by 75% of the initial infusion rate during the first step and 85% of the basal rate during the second step of the clamp to maintain constant plasma glucose enrichment^39^. We used a warming blanket for arterialization of hand vein. Plasma glucose level in arterialized blood was maintained at ~95 mg/dl by a variable 20% glucose infusion containing ~2.5% [6,6-^2^H_2_]glucose. Blood samples were withdrawn for biochemical analysis at 10 min intervals during the last 30 min before the clamp and during the steady state periods of the first and second steps of the clamp. Enrichment of [6,6-^2^H_2_]glucose in plasma was measured by high-performance liquid chromatography (LTQ-XL-Orbitrap mass spectrometer, Therm Scientific, CA) as described previously^10^. A steady state equation was used to calculate the rates of EGP and Rd at each step^40^. EGP and Rd were normalized by BSA and FFM, respectively^10^. We divided % reduction of EGP at the first step by SS_SI_ and used it as an index of hepatic insulin sensitivity^13^. Similarly, Rd at the second step was divided by SS_SI_ and used as an index of muscle insulin sensitivity^12^.

### Calculation of metabolic clearance rate for serum insulin (MCRI)

The MCRI during glucose clamp at the second step was calculated by the following equation^30^: MCRI = {IIR/[SS_SI_ − (B_SI_*SS_SC_/B_SC_)]}, where IIR = insulin infusion rate, SS_SI_ = steady state serum insulin during glucose clamp, B_SI_ = basal serum insulin, SS_SC_ = steady-state serum C-peptide during glucose clamp, B_SC_ = basal serum C-peptide. The C-peptide data were entered to take into account the suppression of endogenous insulin secretion by the exogenous insulin.

### ^1^H-MRS

The IMCL values of the right tibialis anterior (TA) and soleus (SOL) muscles and IHL of segment 6 in the liver were measured by ^1^H-MRS (VISART EX V4.40, Toshiba, Tokyo)^41, 42^. After the measurements, IMCL was quantified by methylene signal intensity (S-fat) using creatine signal (Cre) as the reference and calculated as the ratio S-fat/Cre. IHL was quantified by S-fat with H_2_O as the internal reference, and calculated as a percentage of H_2_O + S-fat [S-fat × 100/(H_2_O + S-fat)]^41, 42^.

### Intra-abdominal and subcutaneous fat areas

Intra-abdominal and subcutaneous fat areas were measured with MRI as described previously^42^. Briefly, T1-weighted trans-axial scans were obtained and intra-abdominal and subcutaneous fat areas at the fourth and fifth lumbar interspaces were measured as described previously by using specific software (AZE Virtual Place, Tokyo, Japan)^42^.

### Statistical analysis

Data are presented as mean ± SD or median (range: 25–75%). To approximate normal distribution, log-transformed values were used in the analysis, as appropriate. Data of two groups were compared by unpaired Student t-test. The relation between MCRI at the second step and various metabolic parameters were assessed by Pearson or Spearman correlation coefficient, as appropriate. All statistical tests were two-sided with 5% significance level.
